# My feet – visible but ignored…’ - therapeutic footwear or surgery – that is the question

**DOI:** 10.1186/1757-1146-3-S1-O27

**Published:** 2010-12-20

**Authors:** Anita Williams, Frank Webb

**Affiliations:** 1 University of Salford, Salford, UK

## 

Therapeutic footwear (TFW) is often provided for people with Rheumatoid Arthritis (RA) for reducing foot pain & increasing patient mobility. However patient dissatisfaction & non-compliance means that it may not be the panacea for foot problems that practitioners presume it to be. The purpose of the study was to explore RA patient’s opinions about their foot problems, foot orthoses (FOs) and footwear

### Method

Following ethical approval 22 people participated in focus groups. Thematic data analysis was employed.

### Results

Foot symptoms & the appearance of feet were one of the most worrying aspects of their disease. However, these were ignored by their GPs & consultants. Some participants had experience of being referred for TFW (n=10) but only 2 were wearing it due to appearance. Those wearing FO’s had restricted choices in their own footwear. There was reluctance by their consultants to refer for foot surgery. Most participants would consider surgery in order to be able to function better and wear reasonable shoes

### Discussion

The participant’s feet were extremely visible to them but ignored by practitioners. Unlike any other intervention TFW replaces something that is normally worn & is part of an individual’s body image. Likewise, the use of foot orthoses impacts on the choice of footwear style. The reluctance of consultant rheumatologists to refer for surgery was of concern, particularly as the participants had knowledge that this would be beneficial even before major structural changes occurred in their feet.

There is a great need for foot surgery to be a management option earlier in order to improve function and reduce pain. Additionally it is a realistic option so that people with RA are not condemned to a life time of wearing footwear that is unacceptable and restricts choice in clothing, reduces participation in activities, particularly social ones. A large multi centre clinical trial is needed that would compare the results of foot surgery versus therapeutic footwear in terms of the clinical and patient orientated outcomes.

